# Advancing responsible genomic analyses of ancient mollusc shells

**DOI:** 10.1371/journal.pone.0302646

**Published:** 2024-05-06

**Authors:** Raphaël Martin-Roy, Jakob Thyrring, Xavier Mata, Pernille Bangsgaard, Ole Bennike, Gunvor Christiansen, Svend Funder, Anne Birgitte Gotfredsen, Kristian Murphy Gregersen, Camilla Haarby Hansen, Peter Carsten Ilsøe, Lutz Klassen, Inge Kjær Kristensen, Gerd Bindesbøl Ravnholt, Frédéric Marin, Clio Der Sarkissian

**Affiliations:** 1 Centre for Anthropobiology and Genomics of Toulouse, UMR5288, CNRS, University Paul Sabatier, Toulouse, France; 2 Department of Ecoscience, Aarhus University, Aarhus, Denmark; 3 Arctic Research Centre, Aarhus University, Aarhus, Denmark; 4 Globe Institute, Section for GeoGenetics, University of Copenhagen, Copenhagen, Denmark; 5 Geological Survey of Denmark and Greenland, Copenhagen, Denmark; 6 Museum of Copenhagen, Copenhagen, Denmark; 7 Natural History Museum of Denmark, Copenhagen, Denmark; 8 Museum Østjylland, Randers, Denmark; 9 Museum Salling, Skive, Denmark; 10 Biogéosciences, UMR6282, CNRS-EPHE-uB, University of Burgundy, EPHE, Dijon, France; Bigelow Laboratory for Ocean Sciences, UNITED STATES

## Abstract

The analysis of the DNA entrapped in ancient shells of molluscs has the potential to shed light on the evolution and ecology of this very diverse phylum. Ancient genomics could help reconstruct the responses of molluscs to past climate change, pollution, and human subsistence practices at unprecedented temporal resolutions. Applications are however still in their infancy, partly due to our limited knowledge of DNA preservation in calcium carbonate shells and the need for optimized methods for responsible genomic data generation. To improve ancient shell genomic analyses, we applied high-throughput DNA sequencing to 27 *Mytilus* mussel shells dated to ~111–6500 years Before Present, and investigated the impact, on DNA recovery, of shell imaging, DNA extraction protocols and shell sub-sampling strategies. First, we detected no quantitative or qualitative deleterious effect of micro-computed tomography for recording shell 3D morphological information prior to sub-sampling. Then, we showed that double-digestion and bleach treatment of shell powder prior to silica-based DNA extraction improves shell DNA recovery, also suggesting that DNA is protected in preservation niches within ancient shells. Finally, all layers that compose *Mytilus* shells, i.e., the nacreous (aragonite) and prismatic (calcite) carbonate layers, with or without the outer organic layer (periostracum) proved to be valuable DNA reservoirs, with aragonite appearing as the best substrate for genomic analyses. Our work contributes to the understanding of long-term molecular preservation in biominerals and we anticipate that resulting recommendations will be helpful for future efficient and responsible genomic analyses of ancient mollusc shells.

## Introduction

Thanks to recent advances, high-throughput DNA sequencing (HTS) can now generate billions of DNA reads in reduced time and costs per base. This has allowed to dramatically extend both the amount and the age of the genetic information recovered from ancient samples, making ancient DNA (aDNA) studies possible at the scale of genomes and of populations with unprecedented temporal resolution (see examples in the review by Orlando et al. [1]). Previous work has shed light on the evolutionary relationships among archaic hominins (e.g., [2,3]), past human migrations and social organization (e.g., [4–6]), demographic trajectories (e.g., [7]), adaptation (e.g., [8]), domestication (e.g., [9]), the evolution of microbiota and pathogens (e.g., [10,11]), and paleo-environments (e.g., [12]).

Breakthroughs in the paleogenomic era have heavily relied on the optimization of wet-lab analysis methods tailored to the two main characteristics of aDNA extracts: one, post-mortem degradation of DNA molecules by high fragmentation and cytosine deamination, two, extensive contamination by exogenous DNA mainly originating from microbial colonisation during deposition and/or handling of the remains [13,14]. A first important step up has been the identification of dense skeletal elements favouring aDNA protection from degradation and contamination [15–17]. Moreover, scaling-up ancient genomic datasets has been enabled by methods fine-tuned to highly degraded aDNA, such as DNA library construction (e.g., [18]) and target enrichment by hybridization capture (e.g., [19]). As for DNA extraction, best performances have been obtained using solid-phase methods, where a chaotropic agent, e.g., guanidinium thiocyanate, disrupts the structure of DNA molecules in solution and facilitates their adsorption to a silica (silicon dioxide) membrane [20–22]. Adding predigestion treatments has further improved endogenous DNA recovery by removing contaminant DNA through multiple digestions of powdered samples [23,24] and/or predigestion washes in sodium hypochlorite (bleach) or phosphate buffers [25–27].

Today, method improvement is not only motivated by dataset age, size or costs, but also by ethical considerations [28,29]. As aDNA analyses (partially) destroy valuable, rare, and sometimes unique and irreplaceable samples, conducting responsible aDNA research and conserving our scientific and bio-cultural heritage has required establishing guidelines: e.g., use of minimally invasive methods [17,30,31], extensive documentation of experimental steps (by laboratory information management systems LIMS) [32], and recording of the samples’ 2D/3D morphological information through photography, photogrammetry, surface scanning or micro-computed tomography (micro-CT) [33–37].

Until now, methodological optimization and standards for ethical research have mostly focused on bones and teeth. Meanwhile, mollusc shells have emerged as promising substrates for DNA analyses (e.g., [38–45]) of samples as old as 100,000 years Before Present (yBP; [46]; reviewed in Martin et al. [47]). In a similar way to morphological, sclerochronological, sclerochemical, or dating methods commonly applied to ancient mollusc shells from shell middens, refuse dumps, sediment cores or historical collections, aDNA analysis has the potential to provide invaluable evolutionary, ecological and archaeological insights into the impact of climate and environmental changes, as well as into past human activities and resource management strategies: subsistence systems, mobility, migration, exchange networks, aquaculture, production of tools and symbolic artefacts (reviewed in Coutellec [48] and Thomas [49,50]). However, only few studies have been conducted at the genomic [46,51,52] and metagenomic scales [53]. One limitation is that the presence of DNA in acellular shells is not fully explained by the currently known cellular mechanisms underlying shell biomineralization: incorporation of outer mantle epithelial cells [47], entrapment of hemocytes [38,54,55], and/or integration of the cellular content of exosomes [56] or that secreted by mantle tissue cells [57]. It has also been brought to attention that genomic data generation from ancient mollusc shells had not been formally optimized [47].

Here, to foster responsible research conduct, we aim at advancing methods for the genomic study of ancient mollusc shells by evaluating the effect of micro-CT scanning on their DNA and by identifying the most performant DNA extraction protocols. To this end, we analysed HTS data from 27 ancient shells dated to ~111–6500 yBP and identified as mussels belonging to the *Mytilus edulis* species complex. Mussels are a relevant model as they are plentiful in the European archaeological and museal records. This is due to *Mytilus* being one of the most abundant genera of the costal animal biomass [58] and a readily collectable resource for alimentary, cultural or scientific purpose from prehistory to present day. Also, in addition to the periostracum, an organic layer covering the outer surface, *Mytilus* shells are composed of two superimposed layers of aragonite and calcite in the inner nacreous and outer prismatic layers, respectively. Since aragonite and calcite are the two main calcium carbonate polymorphs found in mollusc shells [57], conclusions drawn here may be applicable to a vast cohort of representatives for nacro-prismatic molluscs. We also compared DNA recovery from the two carbonate layers with or without the periostracum in order to document future sampling strategies, as shell layers are oftentimes found dissociated in fragmented fossil assemblages. To conclude, we propose recommendations intended to aDNA researchers and collection curators to promote this promising line of research while contributing to the conservation of scientific collections.

## Materials and methods

### Experimental design overview

We investigated methods to optimize the recovery of genomic information from ancient mollusc shells using 27 *Mytilus* specimens selected from archaeological sites and museum collections in Denmark, Greenland and France to represent variation in latitudinal origin (from the Mediterranean Sea to Greenland) and age (~111–6500 yBP based on radiocarbon-dating, archaeological context or collection records; Table 1 and S1 Table).

**Table 1:** Description of the ancient samples analysed in this study.

Specimen Name	Site Name, Country	Latitude (N)	Longitude (W)	Culture; Age	Test (aliquots)
bal05/bal05b	Balaruc-les-Bains, France	43.45	3.69	575–650 C.E.^a^	Digestion (2)/Bleach (3)
bal06				475–500 C.E.^a^	Shell layers (2)
cop01x3	Central Copenhagen Harbor, Denmark	55.68	12.58	Late Medieval period; prior to 1500 C.E.^a^ [59]	Periostracum (2)
cop02				Late Medieval period; prior to 1500 C.E.^a^ [59]	Radiation (4)
cop02x3				Late Medieval period; prior to 1500 C.E.^a^ [59]	Digestion (2)
cop03x3				circa 1630–1680^a^ [59]	Bleach (3)
cop04				circa 1630–1680^a^ [59]	Radiation (4)
cop04x3				circa 1630–1680^a^ [59]	Periostracum (2)
cop05x1L				circa 1750–1820^a^ [59]	Periostracum (2)
cop05x2				circa 1750–1820^a^ [59]	Shell layers (2)
cop06				circa 1570–1620^a^ [59]	Periostracum (2)
fan01	Fannerup F, Denmark	56.39	10.71	Early Ertebølle Culture; 7000–6500 yBP^b^ [60]	Shell layers (2)
hav04	Havnø, Denmark	56.71	10.17	Ertebølle Culture; 7000–5000 yBP^a^ [61]	Radiation (4)
hav05				Ertebølle Culture; 7000–5000 yBP^a^ [61]	Shell layers (2)
hav06				Ertebølle Culture; 7000–5000 yBP^a^ [61]	Bleach (3)
ika01	Ikaasap Ittiva, Southeast Greenland	65.55	−37.12	Thule Culture; circa 1500–1900 C.E.^a^	Digestion (2)
kar21	Kara Sea, nd	75.57	73.31	1882–1883 C.E. (Dijmphna Expedition)^c^	Extraction (3)
nip03	V54 Nipaitsoq, West Greenland	64.08	−50.09	North Culture; circa 1000–1400 C.E.^a^	Extraction (3)
qoo01	Qoornoq, Southeast Greenland	nd	nd	Thule Culture; circa 1700–1900 C.E.^a^	Extraction (3)
rin01	Ringkøbing, Denmark	56.05	8.12	1912 C.E.^c^	Extraction (3)
rin13/rin13b				1912 C.E.^c^	Digestion (2)/Periostracum (2)
rin10				1912 C.E.^c^	Bleach (3)
sis01	Sisimiut—Nipisat, Greenland	66.82	−53.5	6280–6220 yBP^b^ [62]	Bleach (3)
son02/son02b	Sønderhald-II, Denmark	56.41	10.31	500 B.C.E.^a^	Digestion (2)/Radiation (4)
son09				1000 C.E.^a^	Shell layers (2)
umi01	Umiivik, West Greenland	68.53	−52.82	1600–1700 C.E.^a^	Extraction (3)
wgr07	West Greenland	nd	nd	143 yBP^c^	Radiation (4)

Dating method: ^a^Archaeological context; ^b^Radiocarbon-dating; ^c^Museum collection records. C.E., Common Era; B.C.E., Before Common Era; yBP, years Before Present; nd, not determined. The number of aliquots/conditions tested for each specimen is indicated in brackets in the Test column.

**Fig 1:**
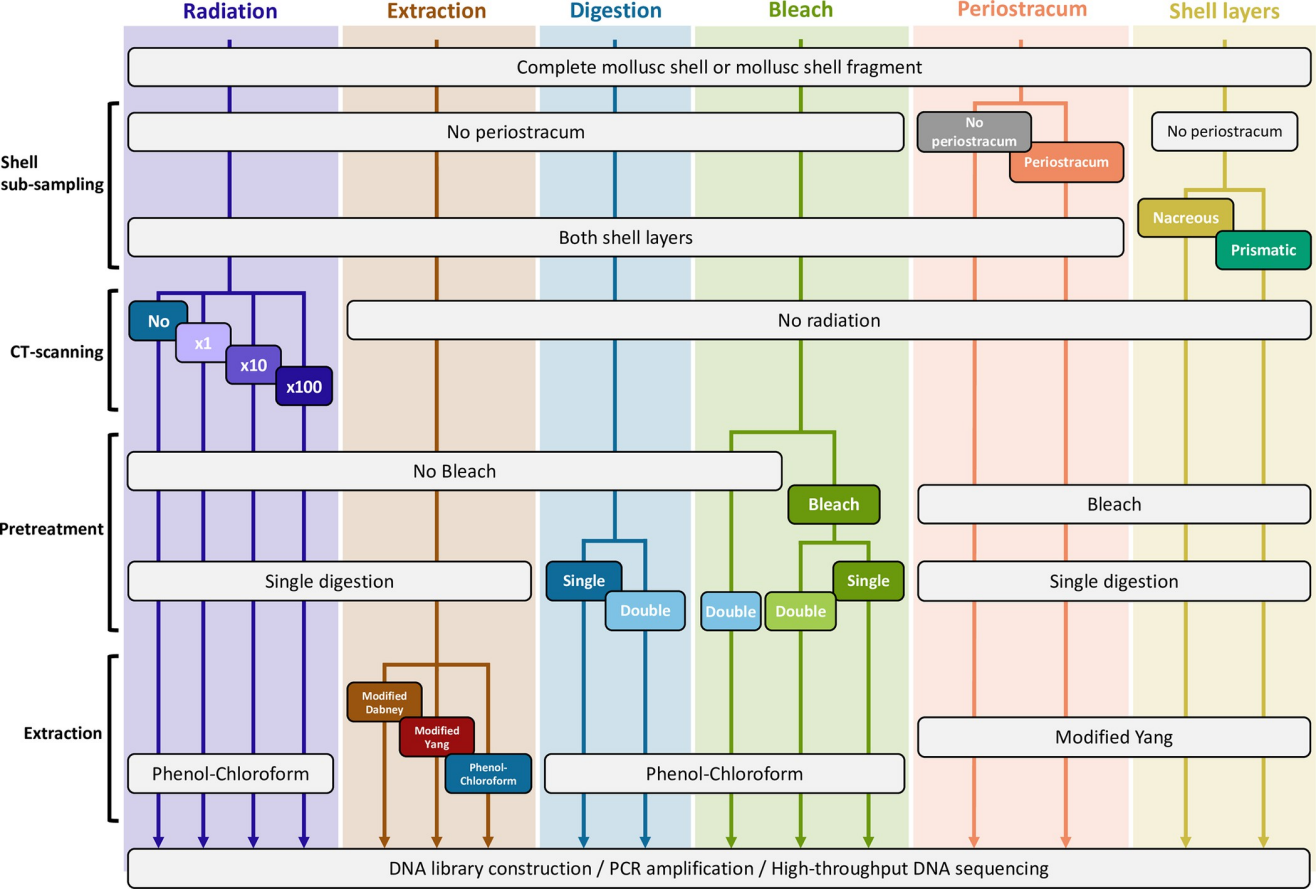
Overall experimental workflow. Experimental conditions were investigated in six main tests: Pre-extraction CT-scanning ‘Radiation’, DNA ‘Extraction’, pretreatments such as ‘Digestion’ and ‘Bleach’ wash, as well as the use of the shell ‘Periostracum’, and the aragonite or calcite ‘Shell layer’ as substrate. Ancient mussel shell DNA recovery was compared in these varying conditions by processing five shells per test, for which a fragment was sub-sampled and powdered before being evenly split into aliquots and subjected to DNA extraction, DNA library construction, PCR amplification, purification and HTS.

All shells were subjected to the same core experimental steps: sub-sampling, DNA extraction, DNA library construction, PCR amplification and purification of libraries, and HTS (Fig 1). For each test, one shell fragment was cut off from five specimens and reduced to a powder that was evenly split into one aliquot per tested condition (Fig 1). Deviating from the core workflow, we first tested whether micro-CT scanning of ancient shells affected their DNA before extraction. Subsequently, we investigated methodological improvements by varying protocols for silica-based DNA extraction, and/or by adding pretreatments. Finally, we examined whether DNA recovery could be improved by retaining the shell periostracum and/or by sub-sampling one or the other calcium carbonate layer constituting mussel shells. Pre-PCR experiments were carried out in isolated laboratories dedicated to aDNA work at the *Centre for Anthropobiology and Genomics of Toulouse* (CAGT, France) following procedures to limit DNA contamination, which was monitored by performing experimental steps (DNA extraction, library construction and PCR set-up) on non-template blank controls, and by sequencing extraction blank controls along shell samples. All experimental steps were documented in our CASCADE LIMS [32].

### Shell sub-sampling

If preserved, and unless stated otherwise, the periostracum was first abraded using a Dremel drill and a sanding tool. A fragment of the shells’ ventral margin was cut avoiding the maximal growth axis so that the rest of the shell could be preserved for future sclerochronological analyses (reviewed in Twaddle et al. [63]). Except for the shell layer extraction test, both shell carbonate layers (inner nacreous and outer prismatic) were kept for DNA extraction. Following Der Sarkissian et al. [53], the ventral margin fragment was decontaminated for 5 minutes under constant agitation in one volume of 1% bleach before being reduced to a powder that was split into aliquots of equal mass (0.13–2.07 g) for direct comparison between conditions for each test (S1 Table).

#### X-ray exposure during CT-scanning (radiation test)

To examine the potential effect of micro-CT scanning, we exposed shell powder aliquots (no periostracum, both shell layers) to: no radiation, the radiation dose emitted during a routine micro-CT scan at ~55 μm resolution, ten and a hundred times the routine dose (Table 1; Fig 1). X-ray experiments were carried out using the EasyTom XL micro-CT scanner (RX solution) at the *Institut de Mécanique des Fluides de Toulouse*, France, with a 1.1 mm-wide aluminium filter placed in front of the source. Then, DNA was extracted using the ‘Phenol-Chloroform’ method described in the section ‘Ancient DNA extraction methods’ below, as it was previously shown to efficiently remove co-extracted inhibitors of subsequent enzymatic reactions [39,42].

#### Ancient DNA extraction methods

We assessed the performance of three silica-based DNA extraction protocols (Table 1; Fig 1). For all methods, shell powder (no periostracum, both shell layers) was digested overnight at 55˚C in 3.85 mL of lysis buffer (0.45 mM EDTA, 0.50% N-lauroylsarcosyl, 0.25 mg/mL proteinase K) under constant mixing. The first protocol (hereafter ‘modified Dabney’) is based on Dabney et al. [20]. The digestion solution was first centrifuged at 1400 xg for 2 minutes, then, the supernatant was mixed with 20 mL of PB buffer (Qiagen) and transferred into a Zymo-Spin V reservoir (Zymo Research) attached to a MinElute (Qiagen) silica column inside a 50 mL tube. This system was centrifuged for 2–4 minutes at 1400 xg until filtration of all the liquid. Subsequent MinElute purification was carried out according to the manufacturer’s instructions. The second protocol (‘modified Yang’) is similar to ‘modified Dabney’ except that, before MinElute purification, the digestion volume was reduced to ~200 μL using an Amicon Ultra-4 30 kD centrifugal filter unit (Merck Millipore) by centrifugation at 1400 xg for 40–50 minutes (Yang et al. [64] as modified by Gamba et al. [22]). The third protocol (‘Phenol-Chloroform’) consists in adding a phenol-chloroform purification step before Amicon concentration in ‘modified Yang’. The digestion volume was thoroughly mixed with one volume (~3.8 mL) of phenol/chloroform/isoamylalcohol (25:24:1) by gentle tube inversion before phase separation by centrifugation at 2000 xg for 10 minutes. The aqueous phase was then transferred to a new tube and the same phenol/chloroform/isoamylalcohol wash was repeated. The resultant aqueous phase was recovered and washed in the same conditions with one volume of chloroform (~3.8 mL). The final aqueous phase was purified using MinElute as in ‘modified Yang’. For all methods, DNA was eluted from the MinElute column after a 10-minute incubation at 37°C using 60 μL of 0.05% Tween in EB buffer preheated at 37°C (‘EB + Tween’; Qiagen).

#### Pretreatments (digestion and bleach tests)

We also evaluated the efficacy of two pretreatments prior to the digestion step. The first pretreatment (‘Double-digestion’; Table 1; Fig 1), is a one-hour predigestion of shell powder (no periostracum, both shell layers) at 37˚C under constant mixing in the same lysis buffer as described in the section ‘Ancient DNA extraction methods’ above [22,23]. Following centrifugation at 1400 xg for 2 minutes, the supernatant was collected and stored at −20˚C. The remaining pellets were resuspended in fresh lysis buffer for an overnight digestion at 55˚C. The second pretreatment (‘Bleach’; Table 1; Fig 1), consists in a wash of the shell powder in 4 mL of 0.5% sodium hypochlorite followed by three washes in 4 mL of molecular biology grade water [25,27]. For samples undergoing one or two such pretreatments, DNA was extracted using the ‘Phenol-Chloroform’ protocol described in ‘Ancient DNA extraction methods’.

#### Periostracum removal and shell layer selection

We next compared DNA yields obtained from two different shell fragments of the same specimen, one with the periostracum removed as described in the section ‘Shell sub-sampling’ above, and the other where the periostracum was kept in place (Table 1; Fig 1). Finally, to determine which of the shell carbonate layers is more suited to aDNA analyses, fragmented shells (Table 1; Fig 1; S1 Fig) were gently crushed using a mortar and pestle to completely separate the layers into two aliquots that were processed as indicated in ‘Shell sub-sampling’. Based on the results of the previous tests, shell powder subsequently underwent the ‘Bleach’ pretreatment and the ‘modified Yang’ DNA extraction protocol for both the ‘Periostracum’ and ‘Shell layer’ tests.

#### DNA library preparation and sequencing

Triple-indexed double-strand DNA libraries for Illumina sequencing were constructed following Fages et al. [65] modified from Rohland et al. [66]. Blunt-ended DNA molecules were first created using the NEBNext End Repair Module (New England Biolabs) with 29.8 μL shell DNA extract in a 50 μL reaction volume and 2.5 U/μL final concentration of T4 DNA polymerase/T4 DNA polynucleotide kinase (incubation at 12˚C for 20 minutes, 37˚C for 15 minutes). Then, two identifying Illumina internal adapters with a 7-bp-index each (10 μM) [66] were ligated at both ends of DNA molecules in 50 μL using and 0.1 U/μL final concentration of T4 DNA ligase (incubation at 20˚C for 20 minutes; NEBNext Quick Ligation Module, New England Biolabs). Finally, the fill-in reaction was carried out in 25 μL using 1.5 U/μL final concentration of New England Biolabs *Bst* polymerase (incubation at 37˚C for 20 minutes and 80˚C for 20 minutes). A MinElute purification was performed after end-repair and ligation, with elution volumes of 30 μL and 20 μL EB + Tween, respectively. Each library was PCR-amplified in 25 μL using 0.4 μL of AccuPrime^TM^ *Pfx* DNA polymerase (Thermo Fischer Scientific), 2.5 μL of 10X AccuPrime^TM^ *Pfx* reaction mix, 4 μL of DNA library, 1 μL of bovine serum albumin (BSA; 20 mg/mL), 15.1 μL of molecular biology grade water, 0.2 μM final concentration of each of the Illumina inPE1.0 and custom 6-bp-index primers as in Fages et al. [65]. Thermocycling conditions were as follows: 95°C for 5 minutes, 12–13 cycles of denaturation at 95°C for 15 seconds, annealing at 60°C for 30 seconds and elongation at 60°C for 30 seconds, followed by a final elongation at 68°C for 5 minutes. Amplified libraries were purified using Agencourt Ampure XP beads (Beckman Coulter) with a 1:1.4 DNA:beads ratio, followed, if necessary, by a second purification with a 1:1 DNA:beads ratio. Library concentration and size distribution were measured on a Tapestation 4200 instrument (High sensitivity D1000 ScreenTapeAssay, Agilent). Libraries carrying a unique index combination were pooled in equimolar proportions and sequenced in paired-end mode (80 cycles) on the CAGT Illumina MiniSeq platform.

#### DNA sequence read processing and alignment

Post-sequencing read processing (de-multiplexing, collapsing and adapter/quality trimming) was performed using AdapterRemoval2 version 2.3.0 [67] and parameters allowing no more than one mismatch per internal barcode (—barcode-mm-r1 1—barcode-mm-r2 1—minadapteroverlap 3—mm 5). Reads were then mapped against the North-European *Mytilus edulis* reference genome (MeduEUN) [68] with PALEOMIX v1.2.13 [69] and BWA version 0.7.15 [70], leaving all parameters as default, except for mapping quality set to 25, and disabling seeding [71]. Duplicated non-collapsed and collapsed reads were removed with MarkDuplicates in Picard Tools version 1.137 and the PALEOMIX FilterUniqueBAM Python script [69], respectively.

#### Taxonomic confirmation

Following the same mapping procedure, we verified taxonomic identifications within the *Mytilus* genus by aligning DNA reads to 208,630 sequences of the 5′-extremity of the mitochondrial cytochrome c oxidase subunit I gene (COI-5P) belonging to the Mollusca phylum in the Barcode Of Life Data System v4 (BOLD; https://boldsystems.org). Here, all the reads obtained for a given sample were analysed together. We only considered reference taxa to which more than 100 bp could be mapped and calculated the percentage of the total mapped bases recovered for each taxon.

#### Protocol performance parameters

To evaluate protocol performance, endogenous DNA content was calculated as the number of unique high-quality ‘mapped reads’ divided by the number of sequencing reads passing quality filters, thereafter ‘retained reads’. Clonality and average mapped DNA fragment length were generated by PALEOMIX. We also investigated possible deviations in GC-content, plus used mapDamage v2 [72] to estimate C-to-T misincorporation rates at the terminal position of the mapped reads’ 5’-end. Finally, we investigated mitochondrial-to-nuclear DNA ratios (corrected by genome sizes) by considering the number of unique high-quality reads aligning to the *Mytilus* MeduEUN nuclear and mitochondrial (Genbank accession number KM192128; 178mc10 in Zbawicka et al. [73]) reference genomes during competitive mapping using the same parameters as described in the section ‘DNA sequence read processing and alignment’ above.

To control for possible biases due to sequencing efforts varying amongst samples for a given test, we calculated all statistics from ten independent random down-samples generated using seqtk version 1.2 (https://github.com/lh3/seqtk). To do this, we first identified, for each test, the sample/condition combination yielding the lowest number of reads post-filtering. Random down-sampling to this minimal number of reads was then performed for the other samples/conditions of the same test. Besides clonality, which is especially susceptible to biases due to sequencing effort variability biases [74] (S2 Fig), we confirmed that all statistical tests led to similar conclusions whether performed on full or down-samples (S2 Table); thus, only clonality results reported in the ‘Results’ section are based on down-samples, while all other results are based on full datasets.

#### Statistical analyses

All statistical analyses were conducted using R v4.3.1 [75]. Normality was assessed using the Shapiro-Wilk normality test (*shapiro*.*test*). Comparisons between treatments were done using either one-sided or two-sided paired t-test (*pairwise_t_test*) or Wilcoxon signed-test (*pairwise_wilcox_test*) when normality was rejected, with functions from the *rstatix* package v0.7.2 [76] and the following parameters: paired = TRUE, p.adjust.method = ‘bonferroni’, alternative = ‘two.sided’, ‘less’ or ‘greater’ (see S2 Table). P-values were calculated with the Bonferroni adjustment method with a significance threshold set to 0.05.

## Results

### Ancient DNA data authenticity

**Fig 2:**
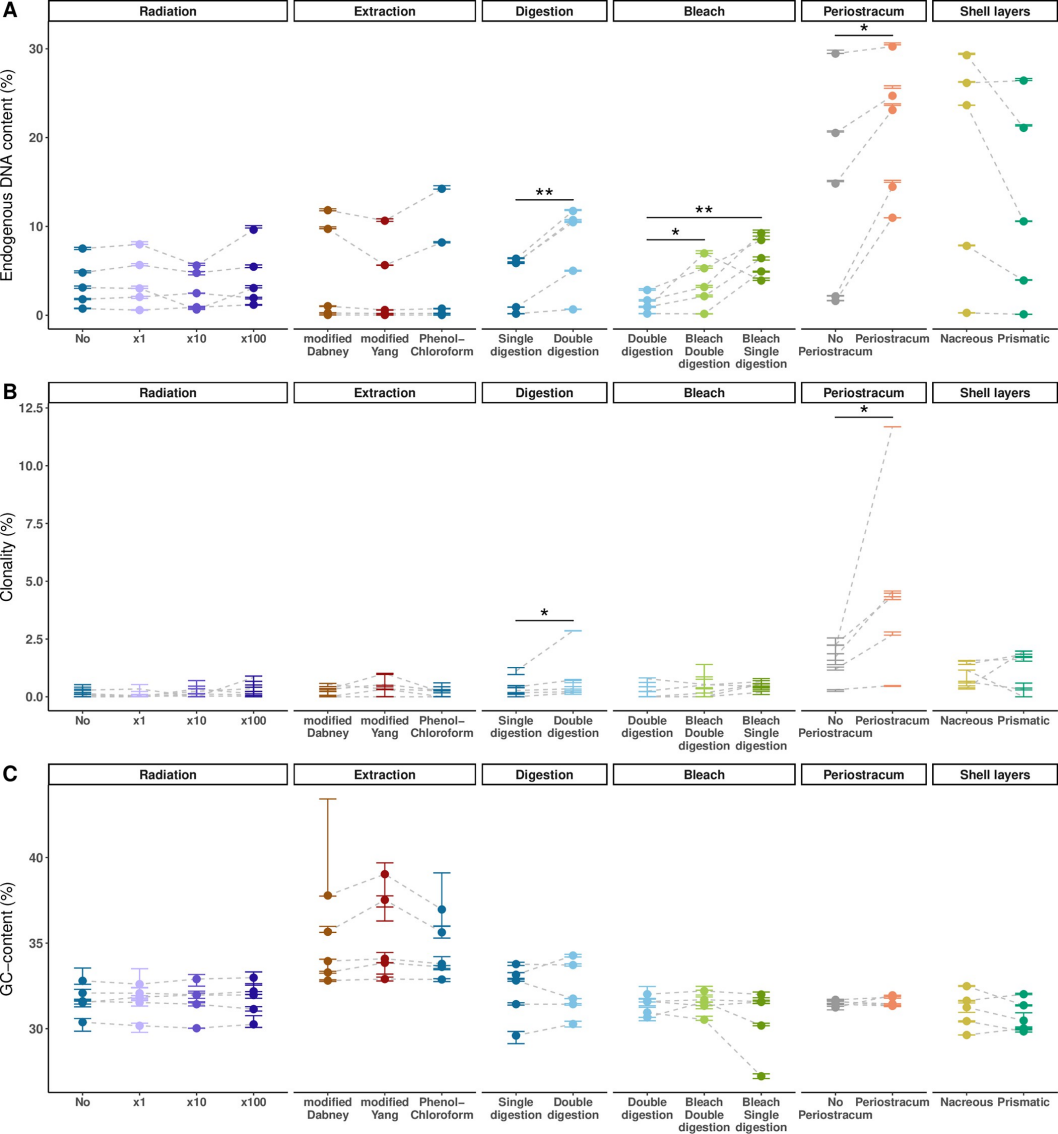
Mapping performances. Ancient shell DNA sequencing reads were mapped to the North-European *Mytilus edulis* nuclear reference genome (MeduEUN) [68]. For a given dataset, parameter estimates calculated from full sequencing datasets are represented by coloured points, error bars correspond to the minimum and maximum parameter estimates calculated from ten down-sampled datasets. Light-grey dashed lines link paired samples (i.e., shell powder aliquots from same sample undergoing different treatments). Statistically significant results are indicated with one (p-value < 0.05) or two (p-value < 0.01) asterisk(s). (A) Endogenous DNA content. (B) Sequence clonality estimated from ten down-samples. (C) GC-content.

We generated shallow HTS data for 80 DNA libraries constructed from 27 ancient shells. In the 22 extraction blank controls sequenced, the maximum value for *Mytilus* sp. DNA content was 0.01% (S3 Fig), which is lower than the 0.03–30% range obtained from shells (S1 Table; Fig 2A). This indicates a low, if any, impact of contamination on our dataset. An additional argument for authenticity is the presence of signatures typical of aDNA in reads mapping to the *Mytilus edulis* nuclear reference genome: increased C-to-T and G-to-A misincorporation rates at the 5’- and 3’-ends of DNA reads, respectively (S4C and S4F Fig) [13], preferential fragmentation after purines (S4B and S4E Fig) [77], and fragment length distributions skewed towards short inserts (average = 69.01 bp; S4A and S4D Fig) [78]. For some samples, length distributions showed a 10-bp periodicity previously explained by nucleosome protection in authentic nuclear aDNA (S4A and S5 Figs), which makes them compatible not only with genomic but also with epigenomic analyses through time [79].

On the basis of COI-5P mitochondrial sequences, we could confirm the taxonomy of all samples, with 82.6–100.0% of the mapped bases aligning to *Mytilus* sp. barcodes (S3 Table). The only false positive molecular identification is for the poorly preserved specimen of unknown age umi01, for which 100.0% of the mapped bases (134 bp) spuriously aligned to sequences from the *Glyptophysa* sp. gastropod (S3 Table). It is highly unlikely that a *Glyptophysa* sp. shell was misidentified as a *Mytilus* sp. shell by collection curators.

### No effect of micro-CT scanning

**Fig 3:**
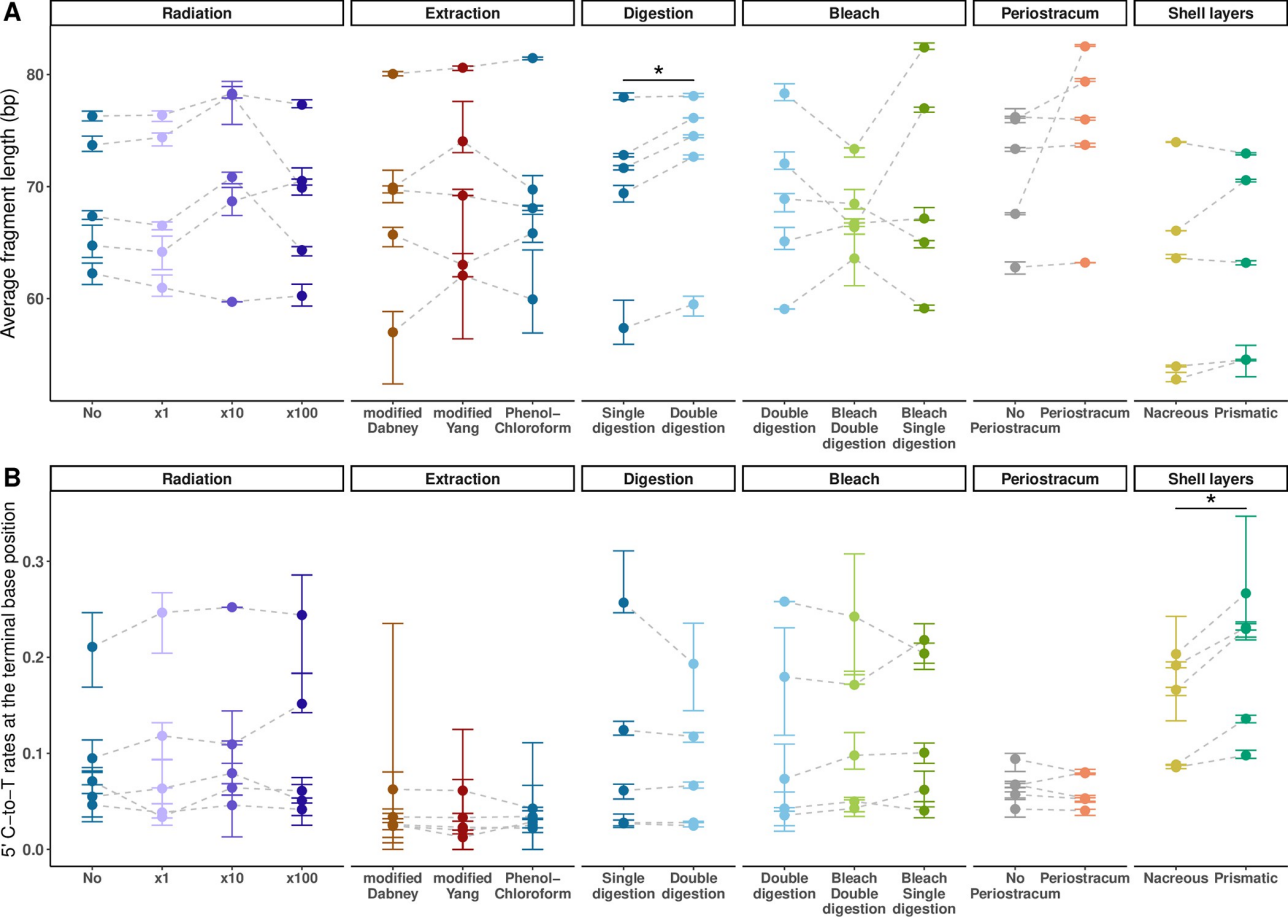
DNA degradation. Ancient shell DNA sequencing reads were mapped to the North-European *Mytilus edulis* nuclear reference genome (MeduEUN) [68]. For a given dataset, parameter estimates calculated from full sequencing datasets are represented by coloured points, error bars correspond to the minimum and maximum parameter estimates calculated from ten down-sampled datasets. Light-grey dashed lines link paired samples (i.e., shell powder aliquots from same sample undergoing different treatments). Statistically significant results are indicated with one (p-value < 0.05) or two (p-value < 0.01) asterisk(s). (A) Average fragment length. (B) C-to-T misincorporation rates at the terminal base position of 5’-ends.

We observed no significant effect of micro-CT radiation exposure across all mapping and damage statistics for all radiation doses, i.e., from no radiation to 100-fold the routine dose (Figs 2 and 3; S2 Table).

### No effect of DNA extraction methods

We next detected no significant differences among the three commonly used aDNA extraction protocols for any of the examined statistics (Figs 2 and 3; S2 Table). Of note, in the case of extremely poor preservation, e.g., for shell umi01, none of the tested method succeeded in salvaging more endogenous molecules. Moreover, we observed increased GC-contents for samples kar21 and umi01, possibly as a consequence of non-specific mapping in conditions of low endogenous DNA content.

### Effect of pretreatments

We then investigated whether DNA recovery could be improved by pretreatments. When compared to single-digestion, double-digestion showed significantly higher endogenous DNA content (paired t-test, thereafter ‘t-test’ = −4.44, df = 4, adjusted p-value, thereafter ‘p-value’ = 0.006; Fig 2A), sequence clonality (paired Wilcoxon signed-rank exact test, thereafter ‘Wilcoxon’, V = 0, p-value = 0.031; Fig 2B) and average fragment length (Wilcoxon, V = 0, p-value = 0.031; Fig 3A) with no effect on GC-content and C-to-T rates (Figs 2C and 3B, S2 Table).

Subsequently, we tested whether adding a bleach wash before single- or double-digestion further improved DNA recovery. Best results were obtained for bleach wash before single-digestion, with significantly higher endogenous DNA content (t-test = 7.11, df = 4, p-value = 0.006) compared to double-digestion alone (Fig 2A), and a trend for higher endogenous DNA content in comparison with the combination bleach wash/double-digestion (S2 Table). Double-digestion was also outperformed by combining bleach wash and double-digestion, with a significantly higher endogenous DNA content (t-test = 2.17, df = 4, p-value = 0.048; Fig 2A). We however detected no significant differences in sequence clonality, average fragment length, GC-content and C-to-T rates across all pretreatments (Figs 2B, 2C and 3; S2 Table).

### Effect of shell sub-sampling

**Fig 4:**
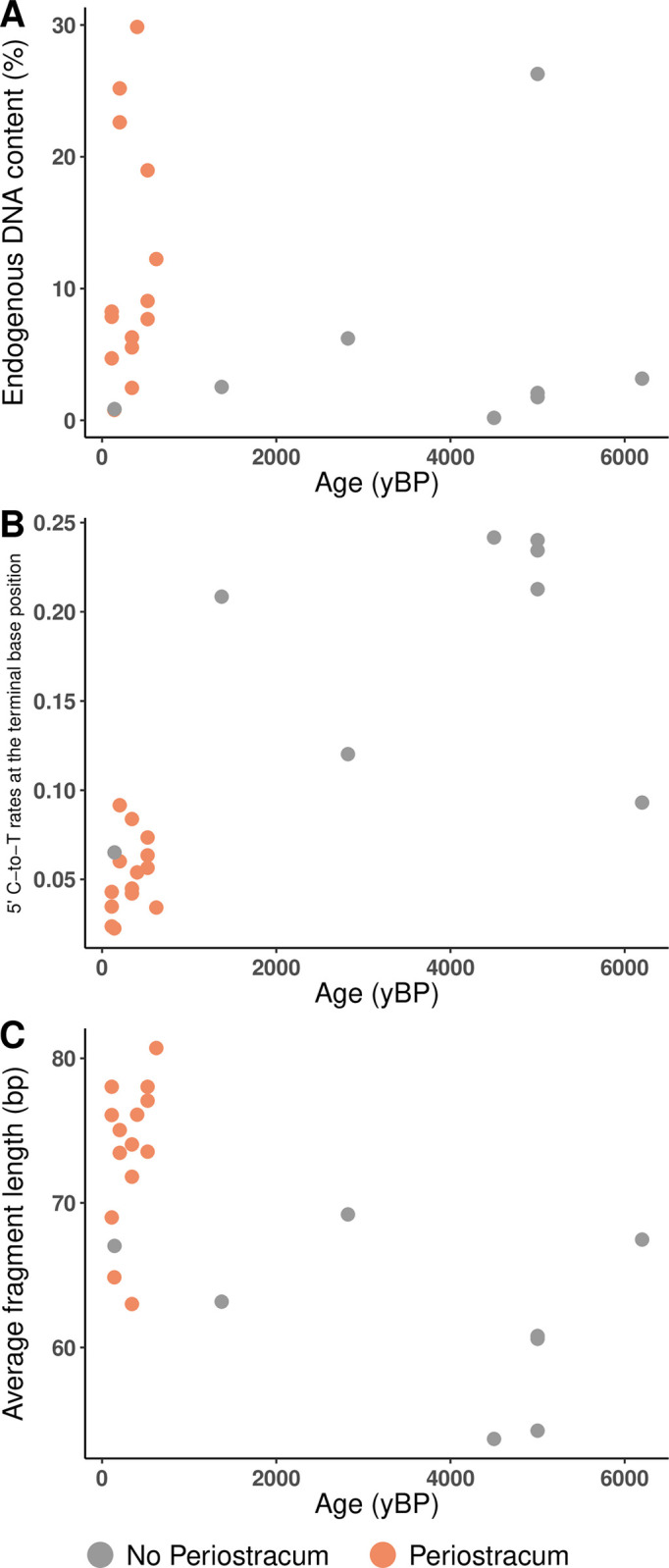
Ancient shell DNA quantity and quality estimates according to specimen age and presence of the periostracum. Each point represents the mean estimate value averaged across conditions for a given dated specimen, with (N = 14) or without a preserved periostracum (N = 8). (A) Endogenous DNA content. (B) C-to-T misincorporation rates at the terminal base position of 5’-ends. (C) Average fragment length.

We then examined whether DNA retrieval could benefit from optimized shell sub-sampling strategies. When retaining the periostracum, higher endogenous DNA content (t-test = −3.45, df = 4, p-value = 0.026) and sequence clonality (Wilcoxon, V = 0, p-value = 0.031) were obtained, with no effect on average fragment length, GC-content and C-to-T rates (Figs 2 and 3; S2 Table). The periostracum was only present on most recent shells (N = 14; ~111 to 623 yBP; Fig 4; S1 Table) for which we confirmed a better DNA preservation as shown by increased endogenous DNA content (Wilcoxon test = 40, p-value = 0.013) and average fragment length (t-test = −4.64, df = 23.8, p-value < 0.001), as well as decreased C-to-T rates (Wilcoxon test = 151, p-value = 0.003) compared to shells without periostracum (N = 13; ~143 to 6500 yBP; Fig 4; S1 Table).

As for the shell layer test, we observed no significant differences across mapping statistics other than higher 5’ C-to-T rates when processing the outer prismatic shell layer (t-test = −4.81, df = 4, p-value = 0.004), which also showed a trend for lower endogenous DNA content (t-test = 1.98, df = 4, p-value = 0.059; Figs 2 and 3; S2 Table).

**Fig 5:**
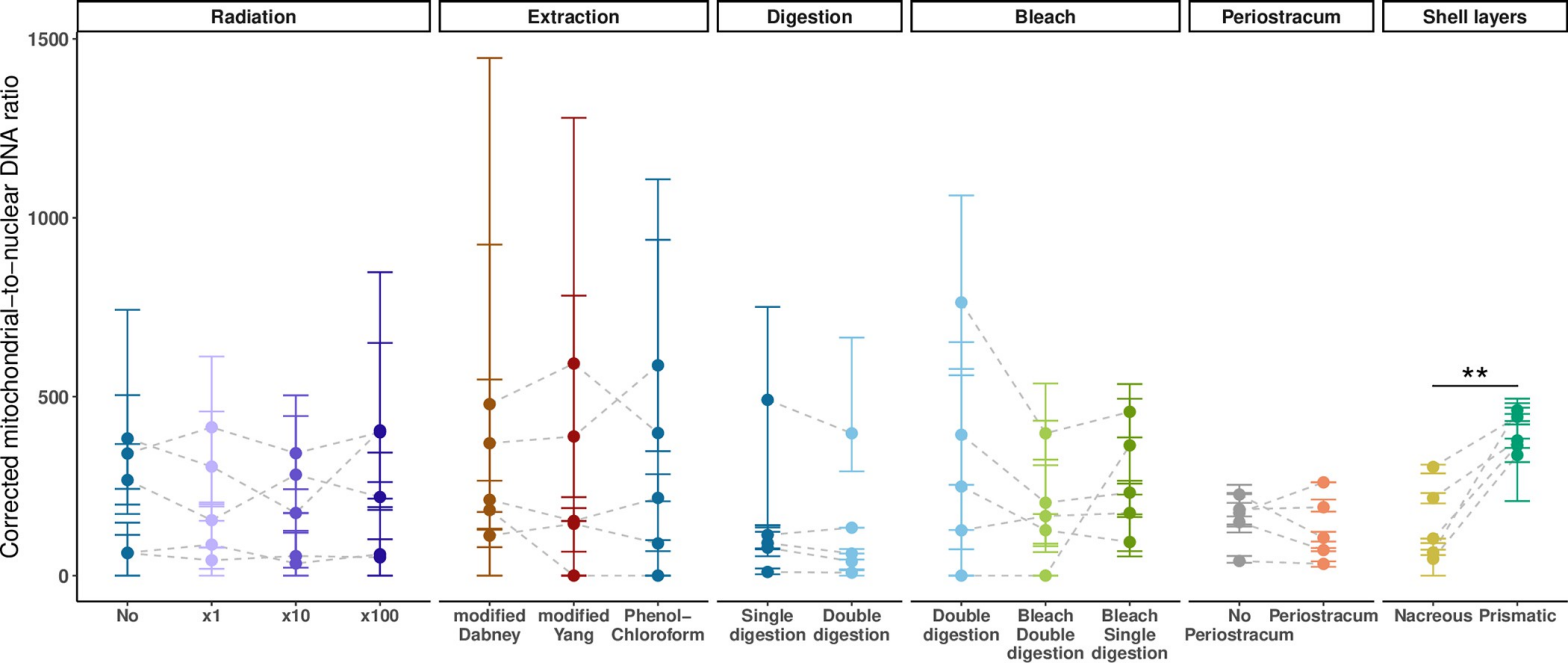
Mitochondrial-to-nuclear DNA ratio corrected by genome sizes. Ancient shell DNA sequencing reads were aligned to the *Mytilus edulis* nuclear (MeduEUN) [68] and mitochondrial reference genomes (178mc10) [73] through competitive mapping. For a given dataset, parameter estimates calculated from full sequencing datasets are represented by coloured points, error bars correspond to the minimum and maximum parameter estimates calculated from ten down-sampled datasets. Light-grey dashed lines link paired samples (i.e., shell powder aliquots from same sample undergoing different treatments). Statistically significant results are indicated with one (p-value < 0.05) or two (p-value < 0.01) asterisk(s).

The shell layer test is also the only test in this study where significant differences in mitochondrial-to-nuclear DNA ratios were found, with the outer prismatic layer showing higher ratios than the inner nacreous layer (t-test = −5.36, df = 4, p-value = 0.006; Fig 5; S2 Table).

## Discussion

On the basis of our methodological tests, we propose guidelines for time- and cost-effective, as well as responsible genomic and epigenomic analyses of ancient mollusc shells.

### Acquire high-resolution 3D images of ancient mollusc shells prior to destructive DNA analyses

Our study demonstrates that micro-CT scanning used to record the 3D morphological information of mollusc shells does not cause any detectable alteration of endogenous DNA. This is the case when subjecting ancient shells to up to 100 times the dose used during routine scanning (~0.2–60 Gray; Gy), in line with the 200 Gy threshold above which lower aDNA quantities and decreased C-to-T rates (likely due to increased fragmentation) were observed in skeletal remains [36]. *In vivo*, it has been shown that lower radiation doses are required to induce DNA damage (10–20 Gy), in the form of altered bases and sugars, single- and double-strand breaks, cross-links and clustering [80]. As these are caused by the release of free hydroxyl radicals and prehydrated electrons during water radiolysis, their impact is limited in dehydrated sub-fossils [80–82]. In absence of cellular DNA repair mechanisms, radiation doses are cumulative, it is therefore absolutely crucial for collection curators to keep a precise record of the specimens’ scanning history (number of scans and acquisition parameters) to avoid exceeding the damaging radiation dose [34,36]. Contrary to cheaper and more-portable imaging techniques (e.g., photogrammetry and structured-light surface scanning), micro-CT appears unaffected by the geometry and aspect of mollusc shells, and we routinely acquire high-resolution scans for 20–30 valves in 12 minutes. In line with ethical guidelines (suggested by Pálsdóttir et al. [37]), we recommend micro-CT scanning of mollusc shells before (partially) destructive sampling, if possible, with a metallic filter placed in front of the source to remove the more damaging low-energy X-rays [36]. Beyond conservation of our scientific and bio-cultural heritage, high-resolution 3D models also provide unique opportunities to study morphological dynamics of mollusc shells through time [83].

### Use silica-based methods for ancient mollusc shell DNA extraction

When comparing three commonly used silica-based protocols for DNA extraction, we observed no significant differences in their performance. Contrary to previous PCR-based results obtained from modern mollusc shells [39,42], there was here no benefit in the extra phenol-chloroform purification step aiming at removing potential inhibitors of downstream enzymatic reactions. Furthermore, phenol-chloroform increases costs (by ~19.5% and ~57% compared to ‘modified Yang’ and ‘modified Dabney’, respectively, in our conditions), hands-on time, user risk (manipulation/storage of toxic chemicals) and equipment requisites (e.g., chemical hood). As in Gamba et al. [22], we found no practical differences in implementing either ‘modified Yang’ or ‘modified Dabney’: the latter, previously applied to ancient shells in e.g., Hayer et al. [41], Psonis et al. [51], Sullivan et al. [52], Walton et al. [44], was nevertheless ~31% cheaper.

### Consider pre-digestion treatments for ancient mollusc shell DNA extraction

All pretreatments led to increased endogenous aDNA contents, with a maximal gain of 500% for bleach wash/single-digestion compared to double-digestion alone. In agreement with Korlević et al. [27] and Boessenkool et al. [25], the bleach wash helped diminish the contaminant fraction in shell extracts, and it even seems to perform marginally better alone than combined with double-digestion, in contrast to results obtained from ancient fish bones by Boessenkool et al. [25]. As for the quality of the recovered DNA, the only improvements brought by pretreatments were for samples that underwent double-digestion, which displayed longer DNA fragments, as previously reported [25,26,84,85]. We also report that double-digestion allowed access to DNA molecules exhibiting reduced C-to-T rates for the most degraded sample in the double-digestion test (1373-year-old bal05). A similar behaviour was observed for the most ancient samples analysed in Ginolhac et al. [26] and Der Sarkissian et al. [24], while Gamba et al. [22] highlighted a sample-dependent impact of double-digestion. Regarding bleach washes, few studies found it detrimental to DNA preservation [21,86], but a majority, including ours, did not retrieve additional aDNA-like damage [25,27,84]. The bleach wash then appears as a safe and efficient way to increase endogenous content in ancient mollusc shell DNA extracts. Some caveats have been expressed though, as pretreatments can result in important losses of endogenous DNA [27], and thus library complexity (higher clonality), which hinders genome coverage at high sequencing depths [74] as observed before [21,23,25]. Considering the variability among samples and experimental set-ups, we would advise to consider pretreatments on a sample-basis, balancing their benefits and drawbacks specifically according to the preservation and quantity available for the sample [21,23], as well as sequencing depth requirements [21]. When applying the double-digestion treatment, the supernatant obtained after the first digestion should be stored to avoid additional shell sampling may a problem occur.

### Use the periostracum as an indicator of good DNA preservation in ancient mollusc shells

We showed that keeping the periostracum increased endogenous DNA content without altering the quality of the DNA recovered from ancient shells, similarly to modern and cooked shells [38,39]. DNA molecules may be incorporated within the periostracum during its secretion by mantle cells at the periostracal groove and may interact with the sclerotized proteins that make up this organic layer [57]; alternatively, the molecules may be trapped between the periostracum and the outer carbonate layer during shell formation [47,54]. The periostracum indeed creates the extrapallial space, a minute cavity between the mantle and the shell sealed from the environment. There, geochemical conditions are maintained to allow shell biomineralization by outer mantle epithelial cells beneath the periostracum that serves as a template for shell growth [57]. In previous work, the periostracum was abraded prior to DNA extraction to remove potential contaminants [42,45], as its high organic content (proteins and polysaccharides) may be prone to microbial colonization, thus reducing the endogenous fraction. However, we observed the opposite here, either because the periostracum is not a good substrate for microbes, or because its chemical compounds prevent bacterial attachment and growth to protect organisms from bacterial fouling [87]. Yet, the post-mortem persistence of these bioactive compounds in ancient shells remains to be investigated. On the downside, keeping the periostracum led to an increase in clonality, which could be due to the co-extraction of molecules inhibiting enzymatic reactions during DNA library construction. Their nature remains hypothetical considering the lack of information on the molecular composition of the periostracum [57]. Consequently, the advantages and drawbacks of adding a phenol-chloroform purification step as in Geist et al. [39] should be weighed against those of removing the periostracum, according to each study’s specific objectives and constraints.

As expected, shells showed better DNA preservation whenever their periostracum was still present. This was the case for the vast majority (14/15) of the most recent shells (within the last ~600 years). Contemporary shells with and without preserved periostracum from similar environments would be required to conclude whether the presence of the periostracum merely is an indicator of good biomolecular preservation or whether it also improves shell DNA preservation. If collectable in a given preservation context, shells having retained their periostracum should be preferred provided that they are radiocarbon-dated or that collections or geological/archaeological layers are temporally defined with confidence to avoid mistaking modern for ancient shells.

### Prefer well-preserved aragonite rather than calcite shell layers for ancient mollusc genomics

Neither the inner aragonitic nacreous nor the outer calcitic prismatic shell layers had a substantial effect on mapping performances, with, however, reduced cytosine deamination rates and a trend for higher nuclear endogenous content in the nacreous layer. The compact microstructure of nacre may provide better protection of DNA against microbial attacks, as well as, hydrolytic and oxidative damage (as shown by lower C-to-T rates), as the nacreous layer is organised in flat aragonitic crystal tablets densely packed in sheets parallel to the shell surface. Conversely, the prismatic layer is less dense and composed of elongated calcite crystals placed perpendicularly or obliquely to the shell surface [57]. In *Mytilus*, prisms are tiny, oblique to the surface and maintained together by organic ‘periprismatic’ sheaths: from a taphonomic viewpoint, this layout is potentially prone to microfluid circulation along prisms axis (i.e., at the interface between neighboring prisms) and colonization by bacteria or fungi. The trend for higher endogenous content in the nacreous layer is reminiscent of the maximised aDNA yields obtained from high-density petrous bones in vertebrates [16] and from aragonitic rather than calcitic mollusc shells [46,53]. Although, the nacreous layer appears as the layer of choice for DNA work, we advise verifying the macroscopical and microscopical integrity of the shells prior to sub-sampling: nacre is the metastable polymorph of calcium carbonate, by opposition to calcite, which is the stable one. Thus, nacre is more prone to recrystallization soon after deposition [57,88] and the impact of such early diagenetic process on DNA is still unknown.

Besides, the nacreous layer showed lower mitochondrial-to-nuclear DNA ratios, thus making it a more optimal substrate for DNA analyses at the nuclear level. As mitochondrial DNA degrades at a slower pace than nuclear DNA under similar conditions due to its smaller size and circularity [85,89], the proportion of mitochondrial DNA in the less protective prismatic shell layer would increase as nuclear DNA degrades at a higher rate. In teeth, mitochondrial-to-nuclear DNA ratios were higher in DNA extracted from cementum and the pulp chamber compared to dentine. Although this could be due to differences in tissue-dependent degradation processes, the cellular mechanisms involved in the formation of dentin could also explain these discrepancies [15]. Unfortunately, lack of thorough knowledge of mollusc shell formation at the cellular level prevents us from further interpretating the differences observed between the nacreous and the prismatic layers.

### Note on the preservation of DNA in ancient mollusc shells

The recovery of DNA after pretreatment suggests that DNA is entrapped deeply within mollusc shells, as previously hypothesized [38,40,46,53] rather than solely adsorbed to the inner and outer shell surfaces in direct contact with the mantle and the periostracum. A deep entrapment (occlusion) of DNA in shells is congruent with recent proteomic data showing that the shell matrix contains not only the ingredient for mineral deposition (in particular acidic proteins with low complexity domains), but also traces of cytoskeletal (actin) and nuclear (histones, histone-like) proteins, suggesting that ‘intracellular’ *sensu lato* components play an unsuspected role in the mineralization process itself [90]. Our results are also in line with the presence of preservation niches in mollusc shells. These were first described microscopically in bones as intergrown crystal aggregates [84] and, later found to protect endogenous DNA against post-mortem water and microbial attack in archaeological bones and teeth [21–27]. DNA is most probably preserved similarly in mollusc shells and skeletal remains, namely by interactions with the macromolecules of the organic matrix, regardless of their nature, proteins or polysaccharides [91], and/or by adsorption to the mineral phase through electrostatic force interactions between the negatively-charged DNA backbone and positively-charged carbonate surfaces [92]. Differences in surface charges between aragonite and calcite could be another explanation for the DNA taphonomic variations observed here between the two carbonate layers. We found that the samples having retained their periostracum and dating to the last ~600 years yielded less damaged DNA molecules in terms of C-to-T misincorporation and fragmentation than older shells. This both supports and challenges previous attempts to characterise temporal trends in mitochondrial DNA preservation from ancient mammal bone, skin and tissue samples over the last 60000 years [93], which found cytosine deamination rates increasing with sample age, but no consistent decrease in fragment length. The number of samples included in our study is too scarce to robustly test for such trends in ancient mollusk shell DNA. However, the depositional/conservation macro- and micro-environments most probably play an important role as exemplified here by the oldest sample displaying C-to-T misincorporation rates and average fragment lengths within the range of those observed for the most recent shells.

Our conclusions about the origin and preservation of DNA in ancient mollusc shells are based on our investigations of *Mytilus* mussel shells, and most likely also apply to other nacro-prismatic genera, such as *Atrina*, *Pinna*, *Pinctada*, and *Unio*. In future work, it would be interesting to compare our results with those obtained from mollusc shells characterised by other microstructures, such as, e.g., crossed-lamellar, granular, homogeneous, or calcitic foliated.

## Conclusions

The presented recommendations will inform decisions by both collection curators and aDNA researchers when granting access to ancient shells and defining optimal aDNA analyses. We anticipate that they will encourage future responsible cross-disclipinary work on ancient mollusc shells and advance this promising line of research. Although our study reinforces previous aDNA results, it is important to keep in mind that these molecular behaviours are described in ancient mollusc shells for the first time here.

## Supporting information

S1 Fig Picture of the ~6500-year-old archaeological mussel shell fan01 before sampling. The inner nacreous and outer prismatic shell layers were separated and extracted independently.
(TIF)

S2 Fig Clonality. Sequence clonality was estimated from alignments to the MeduEUN reference genome [68] using full sequencing datasets for each test, displayed as bars. Error bars correspond to the minimum and maximum parameter estimates calculated from ten down-samples. Samples are ordered by decreasing age from left to right in each panel. For detailed sample information see S1 Table.
(TIF)

S3 Fig Endogenous DNA content in all non-template blank extraction controls. Endogenous DNA content was estimated from alignments to the MeduEUN reference genome [68].
(TIF)

S4 Fig Post-mortem damage patterns in selected samples. Damage was estimated from alignments to the MeduEUN reference genome [68]. Panels A, B and C show damage patterns for a 111-year-old sample, rin02, extracted without the periostracum and using both shell layers (S1 Table). Panels D, E and F show damage patterns for a ~5000-year-old sample, hav05, extracted using the outer prismatic shell layer without the periostracum (S1 Table). (A) and (D) Fragment size distribution. (B) and (E) Base frequency outside and inside the read (represented by the open grey box) of the first ten base pairs from read ends. (C) and (F) Nucleotide misincorporation along the first ten read positions. For detailed sample information see S1 Table.
(TIF)

S5 Fig Fragment length distributions. Samples are ordered by decreasing age from left to right. For detailed sample information see S1 Table.
(TIF)

S1 Table Description of ancient samples used in this study and mapping statistics. (XLSX)

S2 Table Statistical test results. (XLSX)

S3 Table Taxonomic assignment of ancient mussel samples based on mapping to Mollusca mitochondrial COI-5P barcode sequences from the BOLD System v4. (XLSX)
