# Supplementary figures and images for: Advancing responsible genomic analyses of ancient mollusc shells
Source: PLoS One. 2024 May 6;19(5):e0302646. doi: 10.1371/journal.pone.0302646 (PMC11073703; doi:10.1371/journal.pone.0302646)

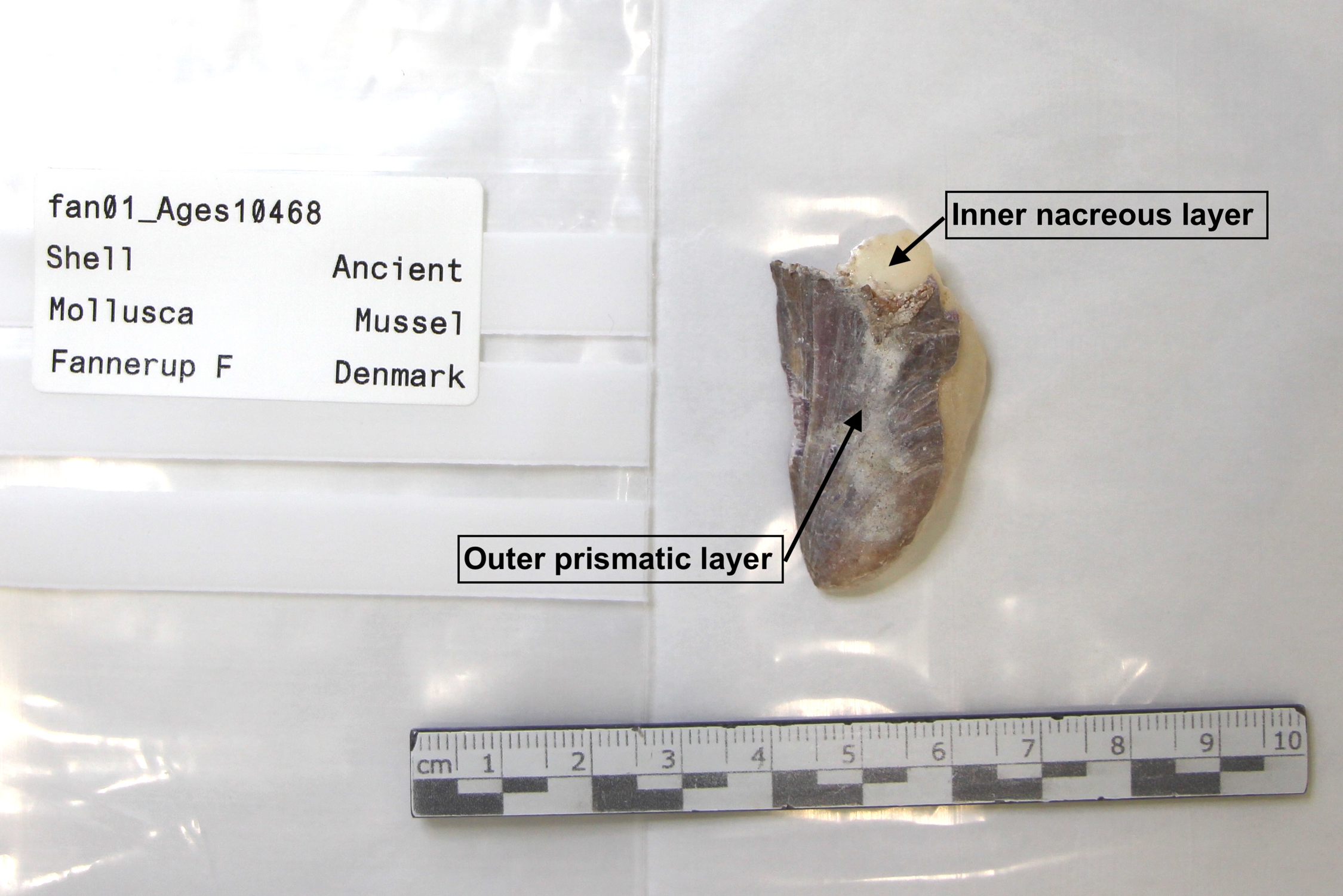

Supplement: S1 Fig — The inner nacreous and outer prismatic shell layers were separated and extracted independently. (TIF) [file pone.0302646.s001.tif]

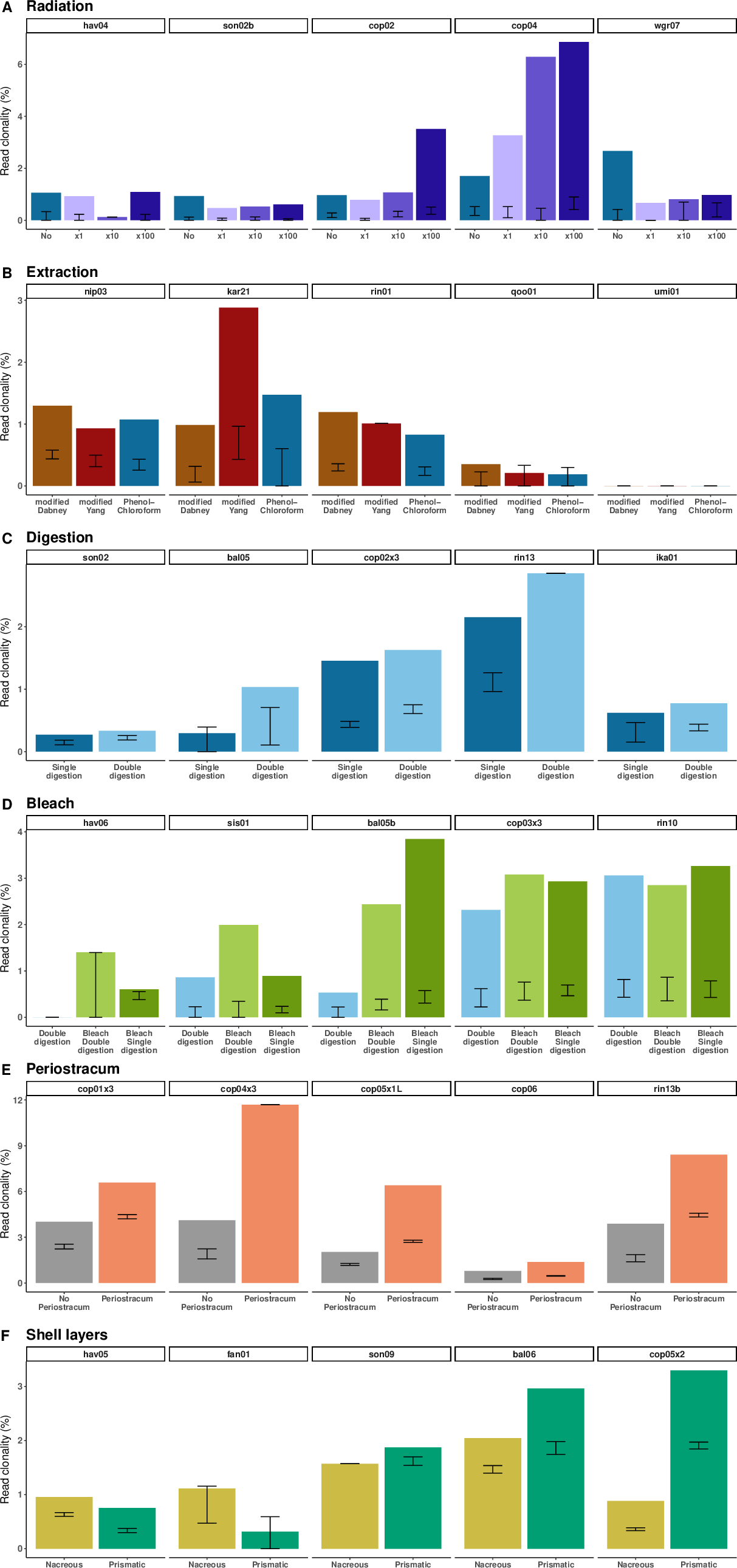

Supplement: S2 Fig — Sequence clonality was estimated from alignments to the MeduEUN reference genome [68] using full sequencing datasets for each test, displayed as bars. Error bars correspond to the minimum and maximum parameter estimates calculated from ten down-samples. Samples are ordered by decreasing age from left to right in each panel. For detailed sample information see S1 Table. (TIF) [file pone.0302646.s002.tif]

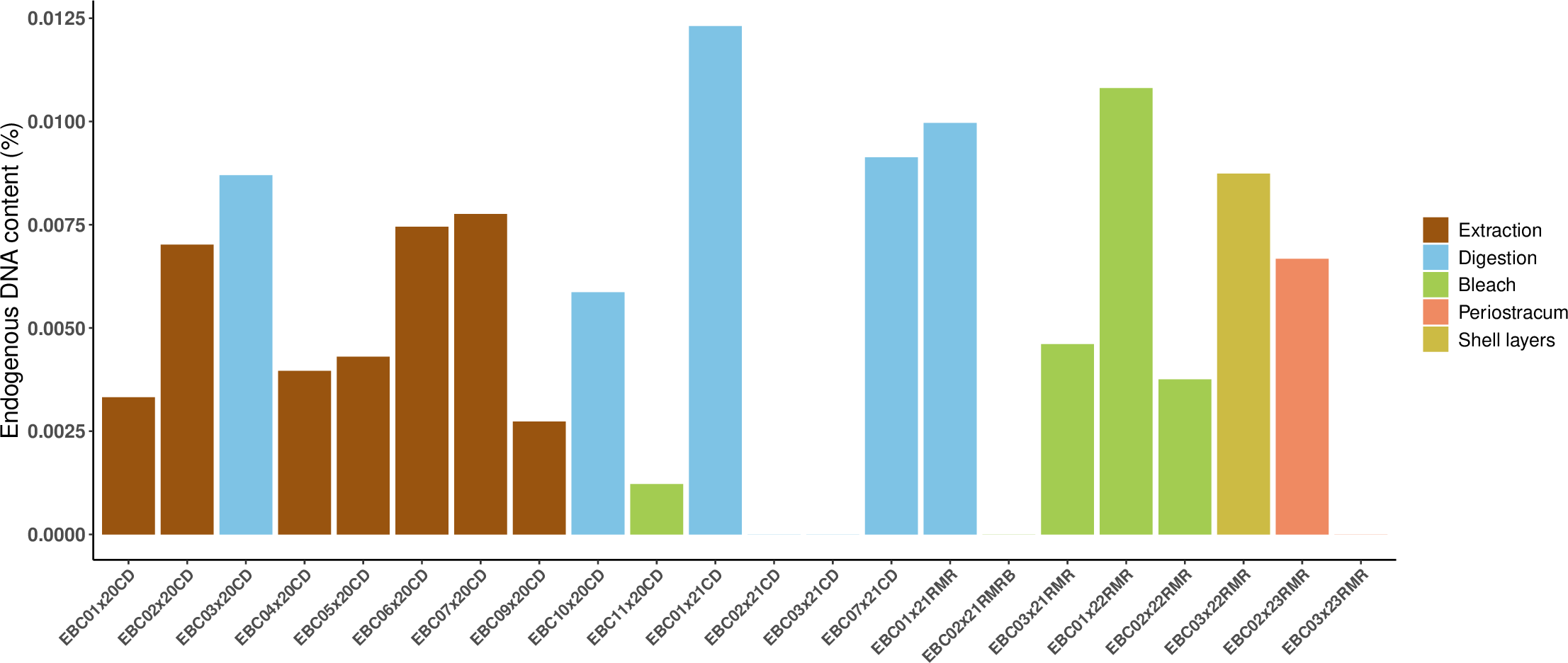

Supplement: S3 Fig — Endogenous DNA content was estimated from alignments to the MeduEUN reference genome [68]. (TIF) [file pone.0302646.s003.tif]

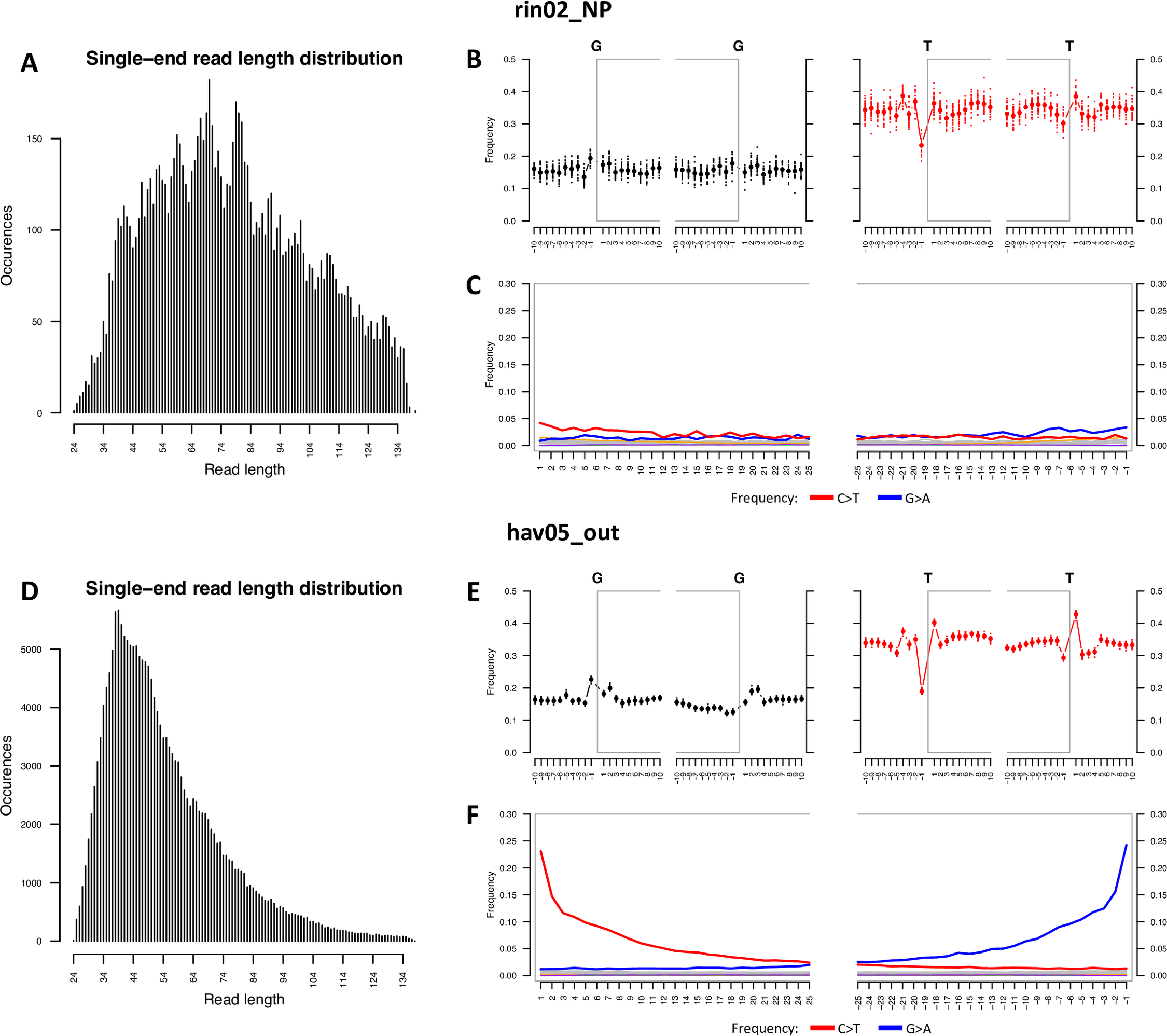

Supplement: S4 Fig — Damage was estimated from alignments to the MeduEUN reference genome [68]. Panels A, B and C show damage patterns for a 111-year-old sample, rin02, extracted without the periostracum and using both shell layers (S1 Table). Panels D, E and F show damage patterns for a ~5000-year-old sample, hav05, extracted using the outer prismatic shell layer without the periostracum (S1 Table). (A) and (D) Fragment size distribution. (B) and (E) Base frequency outside and inside the read (represented by the open grey box) of the first ten base pairs from read ends. (C) and (F) Nucleotide misincorporation along the first ten read positions. For detailed sample information see S1 Table. (TIF) [file pone.0302646.s004.tif]

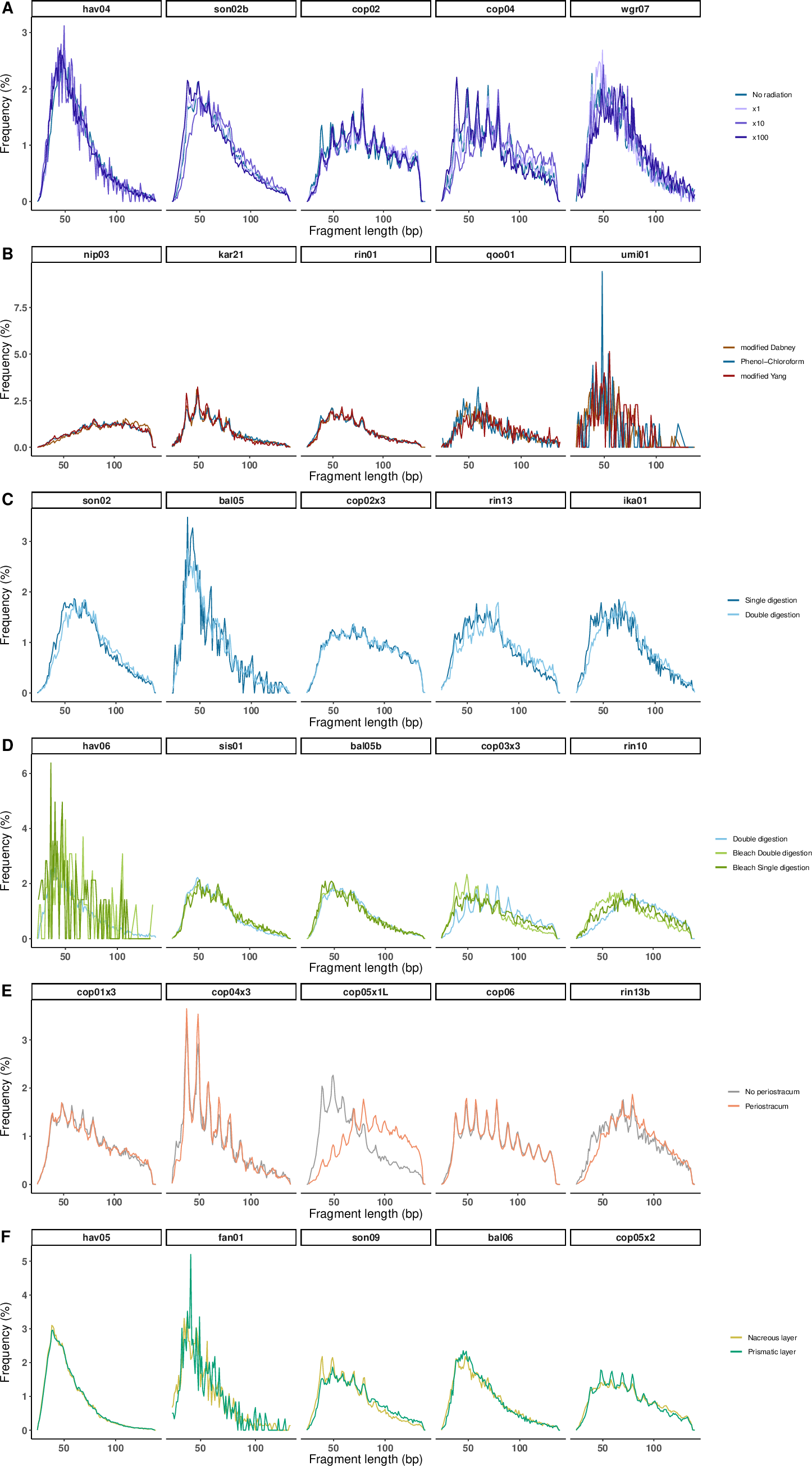

Supplement: S5 Fig — Samples are ordered by decreasing age from left to right. For detailed sample information see S1 Table. (TIF) [file pone.0302646.s005.tif]
